# Multimedia-assisted narrative intervention for existential distress among patients with advanced cancer: a one-group pre–post study

**DOI:** 10.3389/fpubh.2026.1932219

**Published:** 2026-09-15

**Authors:** Qiaoxue He, Xi Huang, Yang Liu, Xiaoqi Li, Lihong Huang, In Hye Park, Yiqiang Guo, Yingchun Chen

**Affiliations:** 1 Integrated Traditional Chinese and Western Medicine Diagnosis and Treatment Center, Siping City Central People's Hospital, Siping, China; 2 Department of Nursing, School of Medicine, Xiamen University, Xiamen, China; 3 School of Nursing, Beijing University of Chinese Medicine, Beijing, China; 4 College of Nursing, Ajou University, Suwon-si, Republic of Korea; 5 Department of Nursing, Kiang Wu Nursing College of Macau, Macau, Macao, China

**Keywords:** advanced cancer, death anxiety, existential distress, meaning in life, narrative intervention, palliative care, public mental health, terminal self-efficacy

## Abstract

**Purpose:**

Patients with advanced cancer often experience challenges related to life meaning, coping confidence, and death-related concerns, which may impair quality of life. This preliminary one-group quasi-experimental pre–post study examined whether meaning in life, terminal self-efficacy, and death anxiety changed after a multimedia-assisted narrative psychosocial intervention. The uncontrolled design was intended to identify preliminary within-participant signals and cannot establish intervention efficacy.

**Methods:**

A one-group quasi-experimental pre–post study was conducted in three hospice care facilities. Of 50 recruited patients, 43 completed the 4-week program and post-intervention assessment. The intervention integrated weekly group storytelling sessions, individual interviews, daily educational dissemination, multimedia-supported reflection, and structured legacy-oriented activities. Outcomes were assessed using validated scales and analyzed with paired-samples t-tests, mean differences, 95% confidence intervals, Cohen's dz, and Holm-adjusted *P* values.

**Results:**

Meaning in life increased from 2.28 ± 0.65 to 2.70 ± 0.52 (mean difference = 0.42, 95% CI 0.33 to 0.51, dz = 1.49), and terminal self-efficacy increased from 2.38 ± 0.58 to 2.88 ± 0.47 (mean difference = 0.50, 95% *CI* 0.40 to 0.59, dz = 1.62). After scale-keying the T-DAS scores so that higher scores indicated greater death anxiety, death anxiety decreased from 3.11 ± 0.27 to 2.97 ± 0.29 (mean difference = −0.13, 95% CI −0.22 to −0.05, dz = −0.49). All Holm-adjusted *P* values for the three primary outcomes were < 0.01.

**Conclusion:**

This preliminary one-group pre–post study observed favorable changes in meaning in life, terminal self-efficacy, and death anxiety following a multimedia-assisted narrative intervention. Because the study lacked a control group and used a small convenience sample, the findings should be interpreted as hypothesis-generating rather than evidence of intervention efficacy.

## 1 Introduction

The International Agency for Research on Cancer reported nearly 20 million new cancer cases globally in 2022, with this number projected to rise to approximately 35 million by 2050 (1). In China alone, new cancer cases reached 4.82 million in 2022 (2). Patients with advanced cancer often experience a multidimensional burden involving severe physical symptoms, fear of death, loss of life meaning, interpersonal detachment, and heightened uncertainty about the future, all of which can substantially impair quality of life and mental health (3). Without systematic psychological support, some patients may resort to maladaptive coping strategies such as denial or self-blame, thereby worsening anxiety, depression, and psychological distress. Existential distress, death anxiety, and loss of meaning among patients with advanced cancer are not only clinical concerns but also important public mental health issues, particularly in hospice and palliative care settings where access to specialized psychosocial services may be limited. Low-intensity and culturally adaptable interventions that can support meaning-making, emotional expression, and coping confidence may therefore have practical value for improving psychological support in end-of-life care.

Palliative care is intended to improve quality of life by addressing physical symptoms, emotional needs, and social connectedness. However, access to palliative care remains limited, and families often assume intensive caregiving responsibilities, which may increase caregiver anxiety and depression and indirectly exacerbate patients' emotional distress and isolation (4). Therefore, alleviating psychological suffering and enhancing meaning in life have become important concerns in contemporary palliative care and public mental health practice (5). In response to these needs, narrative care has emerged as a psychosocial intervention model grounded in narrative medicine, emphasizing the role of personal stories, emotional experience, and sociocultural context in shaping patients' illness trajectories and wellbeing (6). At the end-of-life stage, storytelling and attentive listening to patients' life stories, illness experiences, and psychological concerns may facilitate emotional expression, psychological integration, and a greater sense of peace (7).

From the perspective of narrative medicine and meaning-making theory, structured storytelling, reflective dialogue, peer sharing, and multimedia-supported memory work may help patients organize fragmented illness experiences into coherent personal narratives. Through this process, patients may reappraise the meaning of illness and dying, express unspoken emotions, strengthen interpersonal connectedness, and regain a sense of agency. In Chinese hospice contexts, where death-related communication is often shaped by family protection, emotional restraint, and avoidance of direct discussion about dying, a structured narrative approach may provide a culturally acceptable pathway for indirect but meaningful expression through life review, family messages, legacy materials, and guided reflection. Accordingly, meaning in life, death anxiety, and terminal self-efficacy were selected as three related but distinct outcomes reflecting existentially relevant psychological adjustment near the end of life.

Existing research indicates that narrative care can help patients review and integrate life experiences, express deep emotions, reconstruct self-identity, and reestablish meaning in the face of death (8). However, evidence remains limited regarding structured, nurse-led programs that integrate group storytelling, individual reflection, and multimedia-supported narrative activities in hospice settings (9). Most existing studies have emphasized individualized interventions, with less attention to the potential role of group-based narrative care in fostering emotional resonance and social connection (10). In addition, although digital tools, social media platforms, and multimedia materials offer opportunities to extend psychosocial support beyond conventional face-to-face encounters, their integration into narrative interventions in resource-limited palliative care settings remains insufficiently explored (11). Therefore, this study aimed to conduct a preliminary one-group quasi-experimental pre–post evaluation of a structured multimedia-assisted narrative psychosocial intervention for patients with advanced cancer in Chinese hospice care settings. Specifically, we examined changes in meaning in life, terminal self-efficacy, and death anxiety from baseline to post-intervention among patients with advanced cancer. We expected that patients would show favorable pre–post changes in meaning in life, terminal self-efficacy, and death anxiety after the 4-week intervention compared with baseline.

## 2 Materials and methods

### 2.1 Study design and sample

This preliminary study used a one-group quasi-experimental pre–post design with a convenience sample of patients with advanced cancer and was reported in accordance with the TREND statement for nonrandomized evaluations. A total of 50 patients with advanced cancer were recruited by convenience sampling from three hospice care facilities in Xiamen and Siping, China, between March and May 2024. Given the one-group quasi-experimental design and the limited number of eligible patients in hospice care settings, the sample size was determined pragmatically according to patient availability during the recruitment period, intervention feasibility, and the capacity of the three participating facilities. No formal *a priori* sample size calculation was conducted because this was a preliminary one-group study conducted in hospice care settings. Therefore, the study was designed to provide preliminary estimates of within-participant changes rather than a definitive evaluation of intervention efficacy. Potential participants were identified by clinical staff and screened by the research team according to predefined eligibility criteria. Of these, 43 completed the intervention and post-test assessment, and seven withdrew because of deteriorating health status. Inclusion criteria were: (1) diagnosis of advanced cancer with an estimated life expectancy of less than 6 months; (2) intact cognitive function; and (3) ability to understand the study purpose and procedures and provide written informed consent. Life expectancy and clinical stability were determined by treating clinicians based on routine clinical assessment. Cognitive function was assessed by clinical staff according to routine clinical judgment rather than a standardized cognitive screening instrument. Recruitment was conducted jointly by the research team and clinical staff, ensuring that no other research projects were being conducted simultaneously. Exclusion criteria were: severe cognitive impairment or communication difficulty; unstable clinical condition preventing participation in group or individual narrative activities; severe psychiatric symptoms requiring urgent specialist management; and inability to complete the questionnaires.

### 2.2 Intervention

The intervention consisted of a 4-week structured multimedia-assisted narrative psychosocial program delivered as an adjunct to routine palliative care. It integrated weekly group storytelling sessions, individual semi-structured interviews, daily educational dissemination, multimedia-assisted reflection, and digital social support. The program was designed to facilitate meaning-making and emotional expression by combining emotion-centered narrative care with integrated health education.

The program was delivered by trained research team members with experience in palliative care communication. Before implementation, facilitators received standardized training on narrative interviewing, emotional support, death-related communication, multimedia-assisted reflection, privacy protection, and procedures for responding to participant distress. Intervention delivery was supervised by the research team to promote consistency. Facilitators used standardized group discussion guides and individual interview outlines. Any deviations from the planned intervention process were discussed within the research team.

#### 2.2.1 Daily educational dissemination

Daily educational materials, including videos and written articles, were disseminated through public accounts (official content channels) and group chats (interactive messaging groups) on commonly used social media platforms. To protect privacy, participants were reminded not to disclose identifiable clinical information in group chats. Participants were also informed that posting in group chats was voluntary and that they could choose to share reflections only during individual interviews. Group access was restricted to participants and intervention facilitators, and all shared materials were monitored by the research team. Participants could choose not to post personal reflections publicly and instead share them only during individual interviews. These resources were individually tailored to participants' disease progression and emotional states, covering topics such as meaning-making, life reflection, and acceptance of death. In addition to the educational content, participants were encouraged to share personal experiences, photos, and reflections through the social media platform. To ensure the accuracy and relevance of the shared content, the entire intervention was closely supervised by the research team in collaboration with healthcare professionals.

#### 2.2.2 Weekly narrative group sessions

Each weekly theme included two group meetings and one individual semi-structured interview. Group meetings lasted approximately 60 min, and individual interviews lasted approximately 30 min or were adjusted according to participants' physical condition and willingness to continue. Thus, each participant was expected to receive eight group sessions and four individual interviews over the 4-week intervention period, in addition to daily multimedia-supported reflection and educational dissemination. Attendance and completion of intervention activities were recorded by the facilitators where feasible. During group meetings, participants shared experiences and reflections in a supportive clinical environment, with the aim of enhancing emotional expression, peer resonance, and collective resilience. Individual interviews were used to guide personal life-story reflection and provide emotional support tailored to participants' spiritual and psychological needs.

#### 2.2.3 Narrative Themes and Core Concept

The 4-week intervention followed a structured narrative framework, with each week focusing on a specific theme to guide participants in exploring different aspects of their personal stories and supporting psychological growth. The weekly content included:

(1)**Week 1**: Establishing narrative trust through group introductions and guided recollection of meaningful life moments, including personal milestones, initial emotional responses to diagnosis, and the creation of a “river of life” timeline.

(2)**Week 2**: Facilitating peer story-sharing sessions to foster emotional resonance, mutual understanding, and strength derived from shared experiences.

(3)**Week 3**: Conducting guided writing and group reflection activities centered on life purpose and personal values. Participants were encouraged to express previously unspoken emotions to loved ones through letter writing or audio recordings, promoting emotional release and reinforcing psychological resilience.

(4)**Week 4**: Supporting the creation of a spiritual legacy by recording life insights (e.g., voice messages), crafting legacy artifacts (e.g., family mottos, keepsake recipes), and engaging in collaborative future planning with family members.

To ensure consistency and high quality of content delivered through social media platforms, group sessions, and individual interviews, the research team developed structured educational materials, discussion guides for group meetings, and frameworks for individual interviews. All materials were standardized to align with participants' disease progression and emotional states, ensuring that the intervention content was both responsive to their needs and supportive of the overall objectives. The intervention process and methods are shown in Figure 1.

**Figure 1:**
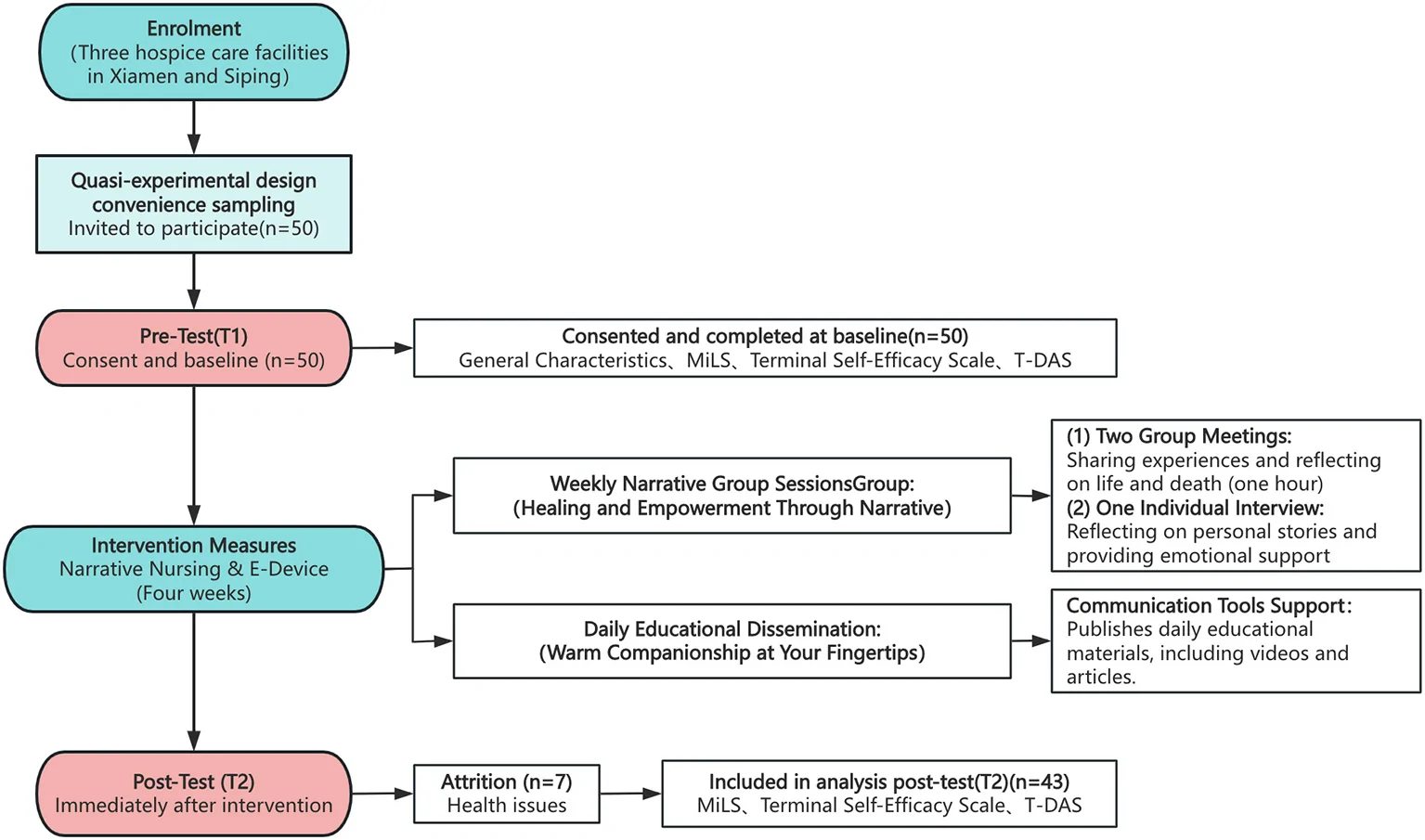
The intervention process and methods.

### 2.3 Measurements

Outcomes were assessed using three validated self-report scales. The primary outcomes were meaning in life, terminal self-efficacy, and death anxiety, measured at baseline and immediately after the 4-week intervention.

#### 2.3.1 General characteristics

Participants self-reported their demographic and socioeconomic information using a structured instrument developed by the research team. Variables included gender, age, diagnosis, duration of illness, household registration, marital status, medical insurance, occupation, education level, hospitalization date, financial stress, and household income. Individual interviews were conducted to ensure participant privacy.

#### 2.3.2 Meaning in Life Scale for Advanced Cancer Patients (MiLS)

Developed by Yongsheng Wu (12), this 28-item scale assesses the sense of meaning and life satisfaction among Chinese patients with advanced cancer. It includes six dimensions: will to find meaning, existential frustration, life purpose, life control, tolerance of suffering, and acceptance of death. Responses are rated on a five-point Likert scale, with higher scores indicating a stronger sense of meaning. The scale demonstrated acceptable internal consistency (Cronbach's α = 0.725).

#### 2.3.3 Terminal Self-Efficacy Scale

Developed by Yaya Kong (13) in 2023, this 22-item scale evaluates terminal self-efficacy across four domains: physical, psychological, spiritual, and social. It uses a five-point Likert scale, with higher scores reflecting greater self-efficacy. The scale demonstrated high internal consistency, with an overall Cronbach's α of 0.916 and domain-specific α values ranging from 0.893 to 0.921.

#### 2.3.4 Templer Death Anxiety Scale (T-DAS)

Originally developed by Templer (14) and adapted into Chinese by Yang (15), the Chinese Likert-type version of the T-DAS includes 15 items. The scale has been reported to have a four-component structure and covers death-related emotional reactions, distress and pain, time awareness, and cognitive concerns. Items are rated on a five-point Likert scale. Items two, three, five, six, seven, and 15 were reverse-keyed according to the scale instructions, and all items were coded so that higher scores indicated greater death anxiety. The total mean score was used as the primary death-anxiety outcome. Subdomain-level scores for emotion, stress and distress, time awareness, and cognition were calculated from scale-keyed item scores and treated as exploratory. The Chinese version has shown good reliability (test–retest reliability = 0.831, Cronbach's α = 0.713); in this study, Cronbach's α was 0.822.

### 2.4 Statistical Analysis

Statistical analyses were conducted using IBM SPSS Statistics version 27.0 (IBM Corp., Armonk, NY, USA). Analyses were conducted among participants who completed both baseline and post-intervention assessments. Descriptive statistics were used to summarize participant characteristics and outcome scores. Continuous variables are presented as means and standard deviations, and categorical variables as frequencies and percentages. For the three primary outcomes, paired-samples t-tests were used to compare baseline and post-intervention scores. Mean differences were calculated as post-intervention scores minus baseline scores. For each primary outcome, 95% confidence intervals, standardized paired effect sizes (Cohen's dz), and Holm-adjusted *P* values were reported. The normality of paired differences was assessed using the Shapiro–Wilk test; when paired differences deviated from normality, Wilcoxon signed-rank tests were planned as sensitivity analyses. Subdomain analyses were exploratory; their P values were not adjusted for multiplicity and were interpreted descriptively. All tests were two-sided, with statistical significance set at *P* < 0.05. Reporting was guided by the TREND statement for nonrandomized evaluations. Given the one-group design, all analyses were interpreted as describing within-participant pre–post changes rather than estimating causal intervention effects.

### 2.5 Ethical considerations

The study was approved by the Ethical Committee of Xiamen University School of Medicine (No. XDYX202304K21). Written informed consent was obtained from all participants before enrollment. To ensure privacy, personal data were anonymized and securely stored in encrypted databases, accessible only to authorized personnel. Because the intervention involved death-related discussion and emotional disclosure, participants were informed that they could pause or withdraw from any activity at any time. If marked emotional distress occurred, the facilitator would stop the activity, provide immediate supportive communication, and refer the participant to clinical staff for further assessment and support.

## 3 Results

### 3.1 Participant flow and baseline characteristics

Of the 50 recruited patients, 43 completed the 4-week intervention and post-intervention assessment and were included in the final completer analysis, yielding a completion rate of 86.0%. Seven patients withdrew because of deteriorating health status before the post-intervention assessment. Baseline characteristics are reported for the 43 completers. Participants who withdrew because of clinical deterioration may have differed systematically from completers in disease severity, symptom burden, or psychological status; however, these differences could not be evaluated because complete baseline information for non-completers was unavailable. Because baseline characteristics of non-completers were not fully compared with those of completers, attrition bias cannot be excluded. Table 1 summarizes the demographic and clinical characteristics of the analytic sample.

**Table 1:** Baseline characteristics of patients with advanced cancer (*N* = 43).

Characteristic	Category	n (%)
Sex	Male	16 (37.2)
	Female	27 (62.8)
Age group (years)	≤ 50	5 (11.6)
	51–60	12 (27.9)
	>60	26 (60.5)
Disease duration	1–3 years	13 (30.2)
	4–6 years	16 (37.2)
	7–12 years	14 (32.6)
Household registration	Urban	35 (81.4)
	Rural	8 (18.6)
Marital status	Married	23 (53.5)
	Non-married	20 (46.5)
Payment of medical expenses	Self-funded	8 (18.6)
	Medical insurance	9 (20.9)
	Publicly funded	26 (60.5)
Education level	Associate degree or above	17 (39.5)
	Secondary school	17 (39.5)
	Primary school or below	9 (21.0)
Admission duration	≤ 3 months	25 (58.1)
	>3 months	18 (41.9)
Monthly household income	< 5000 yuan	14 (32.6)
	5000–9999 yuan	11 (25.6)
	≥10,000 yuan	18 (41.8)

Values are presented as n (%). Percentages may not total 100 because of rounding.

### 3.2 Outcome measures

Table 2 summarizes within-participant pre–post changes in the three primary outcomes and exploratory subdomains among the 43 completers. From baseline to post-intervention, favorable within-participant changes were observed in all three primary outcomes. Meaning in life increased from 2.28 ± 0.65 to 2.70 ± 0.52 (mean difference = 0.42, 95% *CI* 0.33 to 0.51, dz = 1.49, Holm-adjusted *P* < 0.001). Terminal self-efficacy increased from 2.38 ± 0.58 to 2.88 ± 0.47 (mean difference = 0.50, 95% *CI* 0.40 to 0.59, dz = 1.62, Holm-adjusted *P* < 0.001). After scale-keying the T-DAS item scores so that higher scores indicated greater death anxiety, death anxiety decreased from 3.11 ± 0.27 to 2.97 ± 0.29 (mean difference = −0.13, 95% *CI* −0.22 to −0.05, dz = −0.49, Holm-adjusted *P* = 0.003). Exploratory subdomain findings are presented descriptively in Table 2 and were not used to infer primary intervention effects. Because the paired difference for scale-keyed death anxiety deviated from normality, a Wilcoxon signed-rank test was conducted as a sensitivity analysis and yielded a consistent result (*P* < 0.01).

**Table 2:** Pre-post comparison of life meaning, death anxiety, and self-efficacy variables (*N* = 43).

Indicator / Dimensionality	Pre-test (n = 43)	Post-test (n = 43)	Mean difference (post – pre)	95% CI for mean difference	Paired t(df)	Cohen's dz	*P*
	mean ±SD	mean ±SD					
Meaning in life (total)	2.28 ± 0.65	2.70 ± 0.52	0.42	0.33 to 0.51	9.77 (42)	1.49	< 0.001^*^
Will to meaning	2.39 ± 0.78	2.84 ± 0.70	0.45	0.29 to 0.61	5.56 (42)	0.85	< 0.001
Existential frustration	2.22 ± 0.76	2.68 ± 0.77	0.46	0.32 to 0.60	6.58 (42)	1.00	< 0.001
Life meaning and satisfaction	2.31 ± 0.75	2.64 ± 0.63	0.33	0.20 to 0.46	5.07 (42)	0.77	< 0.001
Life control	2.34 ± 0.68	2.77 ± 0.64	0.43	0.27 to 0.59	5.56 (42)	0.85	< 0.001
Endurance of suffering	0.63 ± 0.23	0.73 ± 0.28	0.10	0.03 to 0.17	2.79 (42)	0.43	0.008
Death acceptance	2.20 ± 0.75	2.58 ± 0.75	0.38	0.25 to 0.51	5.75 (42)	0.88	< 0.001
Death anxiety (total)	3.11 ± 0.27	2.97 ± 0.29	−0.13	−0.22 to −0.05	−3.19 (42)	−0.49	0.003^*^
Emotion	3.18 ± 0.50	3.08 ± 0.51	−0.10	−0.29 to 0.09	−1.09 (42)	−0.17	0.282
Stress and distress	2.91 ± 0.41	2.80 ± 0.65	−0.10	−0.25 to 0.04	−1.42 (42)	−0.22	0.162
Time awareness	3.50 ± 0.49	3.13 ± 0.42	−0.37	−0.50 to −0.23	−5.65 (42)	−0.86	< 0.001
Cognition	2.27 ± 0.64	2.62 ± 0.71	0.35	0.19 to 0.51	4.32 (42)	0.66	< 0.001
Terminal self-efficacy (total)	2.38 ± 0.58	2.88 ± 0.47	0.50	0.40 to 0.59	10.64 (42)	1.62	< 0.001^*^
Physiological death	2.31 ± 0.61	2.75 ± 0.66	0.44	0.30 to 0.58	6.52 (42)	0.99	< 0.001
Psychological death	2.46 ± 0.65	2.75 ± 0.66	0.29	0.06 to 0.52	2.52 (42)	0.38	0.015
Spiritual death	2.35 ± 0.75	2.91 ± 0.72	0.56	0.42 to 0.70	8.01 (42)	1.22	< 0.001
Social death	2.39 ± 0.68	3.05 ± 0.61	0.66	0.49 to 0.83	7.96 (42)	1.21	< 0.001

Mean difference was calculated as post-intervention minus baseline. Cohen's dz was calculated as the mean paired difference divided by the standard deviation of paired differences. P^*^ values for the three primary outcomes were Holm-adjusted.

*P* values for exploratory subdomains were not adjusted for multiplicity and should be interpreted descriptively.

Higher scores indicate greater meaning in life, higher terminal self-efficacy, and greater death anxiety.

Death anxiety scores were scale-keyed at the item level before analysis according to the T-DAS scoring direction; negative mean differences indicate reduced death anxiety. Death-anxiety subdomain scores were calculated from the scale-keyed item scores and are reported as exploratory findings. The endurance-of-suffering subdomain is reported using the metric available in the source data.

## 4 Discussion

In this preliminary one-group pre–post study conducted in Chinese hospice care settings, patients with advanced cancer showed favorable within-participant changes in meaning in life, terminal self-efficacy, and death anxiety after a 4-week multimedia-assisted narrative intervention. The findings suggest that narrative-based activities may be associated with favorable within-participant changes in psychological outcomes; however, the uncontrolled design prevents causal attribution.

The observed increase in meaning in life is consistent with the theoretical premise that guided life review and narrative reconstruction may support meaning-making near the end of life. Through structured storytelling and reflection, patients may have been able to reconnect fragmented illness experiences with personal values and life narratives. This interpretation aligns with previous work suggesting that narrative meaning-making can help individuals integrate illness experiences into a more coherent sense of self and life continuity (16). In the present program, the combination of group storytelling, individualized reflection, and multimedia-supported memory work may have provided repeated opportunities for emotional expression and cognitive reappraisal, although the uncontrolled design prevents attribution of change to any single component (17, 18). Future studies using component analyses are needed to determine which elements contribute most to observed outcomes.

After scale-keying the T-DAS according to the scale scoring direction, death anxiety scores showed a modest pre–post decrease. This finding should be interpreted cautiously because the one-group design cannot separate intervention-related change from nonspecific attention, repeated measurement, or changes in clinical condition. Nevertheless, this pattern is theoretically plausible because structured narrative discussion may provide gradual exposure to death-related concerns and support integration of death cognition with personal meaning (19–21).

The pre–post increase in terminal self-efficacy suggests that this outcome may be sensitive to short-term change in the context of structured narrative support. This finding is clinically relevant because terminal self-efficacy reflects patients' perceived capacity to cope with physical, psychological, spiritual, and social challenges near the end of life. However, because the intervention was multicomponent and uncontrolled, the observed change should be interpreted as a preliminary outcome signal rather than as confirmatory evidence of efficacy (22, 23).

### 4.1 Implications and limitations

These findings suggest that multimedia-assisted narrative nursing may be a feasible adjunctive approach for supporting existentially relevant psychological outcomes; however, the findings should be interpreted as preliminary and hypothesis-generating. The results should be interpreted cautiously because this study used a one-group quasi-experimental pre–post design rather than a randomized or controlled efficacy trial.

Several limitations must be acknowledged. First, this quasi-experimental study was constrained by its small convenience sample (*N* = 43) and the absence of a randomized or concurrent control group, which limits causal inference and generalizability. The design cannot isolate intervention-related change from regression to the mean, nonspecific attention effects, repeated testing, spontaneous fluctuation, or other aspects of routine care. Second, outcomes were assessed only at baseline and immediately after the intervention, so no conclusions can be drawn about the durability of changes over time. Third, seven participants withdrew because of clinical deterioration. Because baseline characteristics of non-completers were not fully compared with those of completers, attrition bias cannot be excluded. Participants who withdrew may have differed from completers in disease severity, symptom burden, or psychological status. Fourth, the intervention bundled group storytelling, individual interviews, daily educational dissemination, multimedia reflection, peer support, and facilitator attention; therefore, the active components cannot be identified. Future component-analysis or dismantling studies are needed to identify the active elements of the intervention. Finally, subdomain analyses were exploratory and should be interpreted descriptively because they were not adjusted for multiplicity.

Future research should use larger controlled or randomized designs with longer follow-up, prespecified implementation outcomes, and clearer assessment of adherence, engagement with multimedia components, intervention burden, and potential adverse emotional responses. Future studies should also examine whether demographic and clinical characteristics influence patients' responses to narrative nursing, but such subgroup or effect-modification analyses require larger samples and should be prespecified (24).

### 4.2 Relevance for clinical practice

These preliminary findings suggest that structured narrative activities may be a promising adjunct to routine palliative care, particularly when integrated with low-intensity multimedia support. For future implementation, clinical teams would need a standardized intervention manual, facilitator training in narrative communication and emotional risk management, clear procedures for digital privacy protection, and criteria for identifying patients who are sufficiently stable and willing to engage in death-related reflection. Implementation may also require protected staff time, appropriate group space, access to digital devices and connectivity, supervision procedures, and consideration of barriers related to staffing availability, digital access, privacy regulation, and cultural reluctance to discuss death. Controlled trials are needed before routine implementation can be recommended.

## 5 Conclusion

This preliminary one-group quasi-experimental pre–post study suggests that participation in a structured multimedia-assisted narrative intervention was associated with favorable within-participant changes in meaning in life, terminal self-efficacy, and scale-keyed death anxiety among patients with advanced cancer in hospice care settings. Given the absence of a control group, small convenience sample, completer-only analysis, and lack of follow-up, these findings should be interpreted as hypothesis-generating rather than as evidence of intervention efficacy. Future randomized or controlled quasi-experimental studies with larger samples, longer follow-up, prespecified implementation outcomes, and assessment of intervention fidelity are needed to confirm effectiveness, identify active components, and evaluate feasibility in diverse palliative care settings.

## Data Availability

The datasets generated and/or analyzed during the current study are not publicly available due to privacy and ethical restrictions related to patients receiving end-of-life care. De-identified data may be available from the corresponding author upon reasonable request and subject to institutional and ethical approval.
